# The Impacts of Vertical Off-Centring, Localiser Direction, Phantom Positioning and Tube Voltage on CT Number Accuracy: An Experimental Study

**DOI:** 10.3390/jimaging8070175

**Published:** 2022-06-21

**Authors:** Yazan Al-Hayek, Kelly Spuur, Rob Davidson, Christopher Hayre, Xiaoming Zheng

**Affiliations:** 1 School of Dentistry and Medical Sciences, Faculty of Science and Health, Charles Sturt University, Wagga Wagga, NSW 2650, Australia; kspuur@csu.edu.au (K.S.); chayre@csu.edu.au (C.H.); xzheng@csu.edu.au (X.Z.); 2 Department of Medical Imaging, Faculty of Applied Health Sciences, The Hashemite University, Zarqa 13133, Jordan; 3 School of Health Sciences, Faculty of Health, University of Canberra, Canberra, ACT 2601, Australia; rob.davidson@canberra.edu.au

**Keywords:** vertical off-centring, localizer direction, phantom positioning, tube voltage, CT number accuracy, radiation dose

## Abstract

Background: This study investigates the effects of vertical off-centring, localiser direction, tube voltage, and phantom positioning (supine and prone) on computed tomography (CT) numbers and radiation dose. Methods: An anthropomorphic phantom was scanned using a Discovery CT750 HD—128 slice (GE Healthcare) scanner at different tube voltages (80, 120, and 140 kVp). Images employing 0° and 180° localisers were acquired in supine and prone positions for each vertical off-centring (±100, ±60, and ±30 mm from the iso-centre). CT numbers and displayed volume CT dose index (CTDIvol) were recorded. The relationship between dose variation and CT number was investigated. Results: The maximum changes in CT number between the two phantom positions as a function of vertical-off-centring were for the upper thorax 34 HU (0° localiser, 120 kVp), mid thorax 43 HU (180° localiser, 80 kVp), and for the abdominal section 31 HU (0° localiser, 80 kVp) in the prone position. A strong positive correlation was reported between the variation in dose and CT number (r = 0.969, *p* < 0.001); 95% CI (0.93, 0.99). Conclusions: Patient positioning demands an approach with a high degree of accuracy, especially in cases where clinical decisions depend on CT number accuracy for tissue lesion characterisation.

## 1. Introduction

The contribution of computed tomography (CT) to the effective dose of patients has continued to increase since the introduction of CT in 1971 [1]. Over the years, continuous technological advances have been at the forefront of optimising radiation dose while maintaining diagnostic image quality. This aim has been achieved by utilising tools, such as automated tube voltage selection (ATVS), iterative reconstruction techniques, adaptive beam collimation in combination with the bowtie filter, and automatic tube current modulation (ATCM) [2,3,4,5].

Based on a target image quality, the ATCM is used to adjust the tube current using the CT localiser images accounting for differences in attenuation; this enables optimization between image noise and radiation dose [6]. The role of the bowtie or beam shaping filters is to allow the maximum beam intensity to impact the thickest part of the patient whilst reducing X-ray intensity at peripheral areas where patient attenuation decreases [7]. However, optimal use of a bowtie filter and ATCM techniques ultimately requires the patient to be optimally centred in the CT scanner at the gantry iso-centre [8]. Improper patient centring will also affect the radiation dose by impairing the functionality of the ATCM based on the localiser size [9].

It is widely accepted that patient off-centring is a factor that impacts the CT number (or Hounsfield Unit (HU)) accuracy [10]. Vertical off-centring of 95% of patients undergoing chest and abdominal CT examination has been reported [11]. The risk of patient off-centring is that the resultant CT number variability will affect the sensitivity and specificity of the CT scan [12]. The CT number accuracy is particularly important with regards to tissue lesion characterisation, whereby relying on this absolute CT number is used for diagnostic and therapeutic purposes [13]. This is relevant in several clinical applications, such as employing the Agatston score to determine the extent of coronary atherosclerosis [14]. The importance of CT number accuracy is also evident upon considering a quantitative evaluation of adrenal masses. For these lesions, a biopsy is recommended with a density greater than 10 HU on an unenhanced CT, while no further investigations are deemed to be necessary when the value is equal to 10 HU or below as the lesion is usually considered as an adenoma [15]. Likewise, a 15 HU threshold in contrast-enhanced CT examination is employed to characterise renal cysts. Increased uptake that increases the value above this threshold would be suggestive of malignancy, while cysts with a density less than 10 HU are more likely considered benign [16]. The importance of CT number accuracy means that the use of CT numbers as absolute values for quantitative evaluation has to be supported with optimal imaging techniques. Furthermore, quantitative information (i.e., Radiomic features) could be extracted from CT images to monitor treatment response in oncology [17]. However, the establishment of standarised imaging parameters (such as the voxel size and the number of gray levels) is required for robust radiomic feature validation and quantitative image analysis [18,19].

This study investigates the effects of vertical off-centring, localiser direction and phantom position on CT numbers and volume CT dose index (CTDIvol). Correlations between radiation dose and CT number change were also investigated due to the few published work in this area.

## 2. Materials and Methods

### 2.1. Phantom and CT Protocol

The study reported here represents a further analysis of data collected as a part of a larger study, which details the experiment and dose measurements [20]. The trunk of a PBU-60 anthropomorphic phantom (Kyoto Kagaku Co., Ltd., Kyoto, Japan) representing an average-sized adult was scanned using a 128-slice MDCT system (Discovery CT750 HD, GE Healthcare). Helical scans were acquired using the following recommended manufacturer scan parameters for an adult of similar size: three tube voltages (80, 120, and 140 kVp) were applied, Smart mA (minimum mA of 15, maximum mA of 625, noise index of 21), 5 mm slice thickness with detector configuration of 46 × 0.625, large bowtie (shaping) filter, 50 cm field of view (FOV), tube rotation time 0.5 s, and 1.375 pitch.

The phantom was scanned from above the lung apices to the level of the anterior superior iliac spine (ASIS). Scans were repeated at six different vertical table heights (±100, ±60, and ±30 mm) and measured from the gantry iso-centre whilst maintaining all other scanning parameters. All measurements were performed using 0° and 180° localisers with the phantom in both supine and prone positions.

### 2.2. CT Number and Dose Measurements

**Figure 1:**
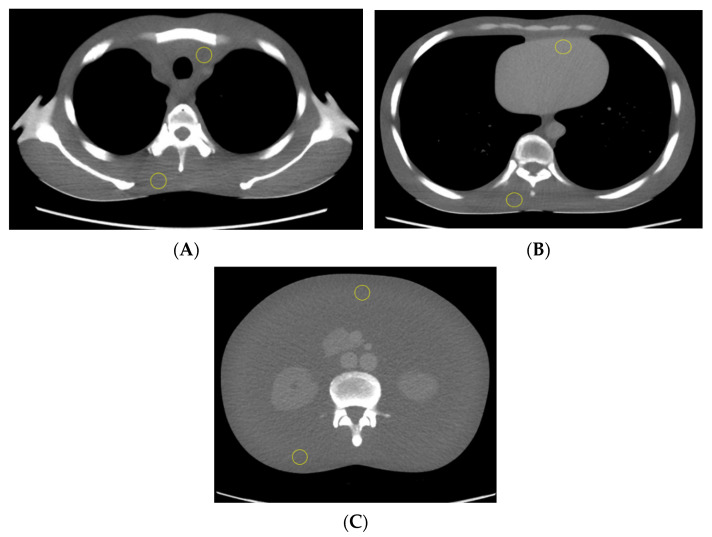
The phantom trunk is pictured supine with axial CT slices of the upper thorax (**A**), mid thorax (**B**), and abdomen (**C**). Two ROIs (yellow circle) were used to calculate the mean CT number measurements.

CT number values were recorded and averaged for three consecutive axial CT slices of the scanned phantom trunk in the upper thorax, mid thorax, and abdomen, as shown in Figure 1. Two regions of interest (ROIs) were plotted on superior and inferior regions in the selected image slice and used to measure the mean CT number in both supine and prone positions. The average CT numbers were measured using ImageJ software [21] and plotted against the phantom vertical off-centring with three tube voltages.

The volume CT dose index (CTDIvol) was recorded with each vertical off-centring increment and experiment configurations.

### 2.3. Statistical Analysis

Statistical analysis was performed using Statistical Package for Social Sciences (SPSS), Version 27 (IBM Corp. Released 2020. IBM SPSS Statistics for Windows, Version 27.0. Armonk, NY, USA: IBM Corp). One-way analysis of variance (ANOVA) was performed to measure statistical difference across phantom vertical off-centring, scanned region, and tube voltage on CT numbers. A paired-samples *t*-test was used to determine the statistical difference in CT numbers between the phantom positions (supine vs. prone) and between the localisers’ direction (0° vs. 180°) as a function of vertical off-centring. A Pearson’s correlation test was used to assess the relationship between radiation dose (CTDIvol) and CT number change as a function of phantom vertical off-centring at different localiser directions and phantom positions. A *p*-value of <0.05 was considered statistically significant.

## 3. Results

Vertical off-centring (above and below gantry iso-centre) was demonstrated a significant influence on CT number, F(6, 245) = 10.35, *p* < 0.001. A Tukey post hoc test revealed that the measured CT number at the gantry iso-centre was statistically significantly changed at +100 mm (14.07 ± 17.56) and −100 mm off-centring (−17.38 ± 18.21). The CT number accuracy was variably influenced based on the scanned region of the phantom, localiser direction, tube voltage and phantom position, as seen in Table 1.

**Figure 2:**
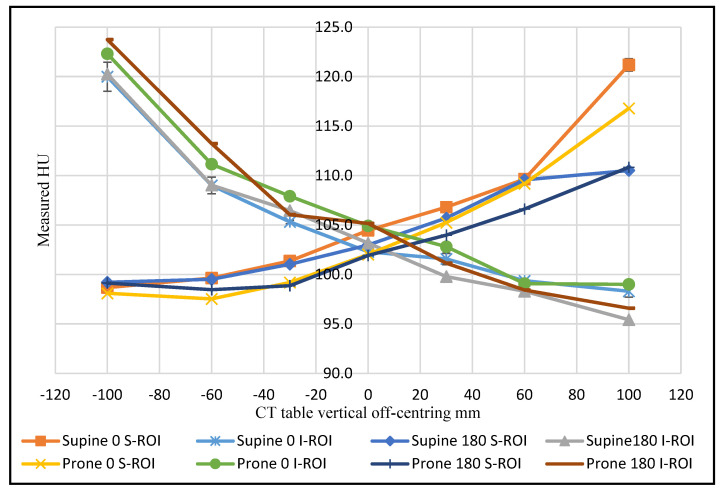
The CT number change as a function of CT table vertical off-centring for the superior and inferior ROIs for the abdominal section at 120 kVp.

As the phantom moved above the gantry iso-centre in the abdominal region, the superior ROI exhibited an increased CT number, coupled with a decrease in the inferior ROI. In other words, an increase in the HU value was recorded when the ROI moved farther from the centre of rotation, and a decrease in HU value was recorded when the ROI was placed closer to the gantry iso-centre shown in Figure 2. With incremental table off-centring, the superior ROI recorded a maximum CT number value of 121.2 HU when the phantom was supine, and the 0° localiser was utilised. The inferior ROI recorded a maximum CT number value of 123.8 HU when the 180° localiser was utilised, and the phantom was prone.

The findings have demonstrated that the use of 0° localiser in combination with vertical off-centring has a significant effect on CT number (M = 0.52, SD = 21.51) compared to 180° localiser (M = −0.63, SD = 21.69); t(125) = 3.44, *p* < 0.001. The CT number was altered for the superior ROI compared to ROI measurements at the iso-centre by 17 HU at +100 mm incremental table off-centring, supine phantom position, whilst utilising the 0° localiser. However, this difference was reduced to 8 HU when the 180° localiser was utilised at the same phantom position.

The findings in the abdominal region also showed a 4.4 HU change for the same superior ROI as a function of phantom position at +100 mm vertical off-centring using a 0° localiser radiograph. The inferior ROI behaved in the same way when the phantom was moved −100 mm from the iso-centre, recording a higher value in the prone position with a difference of 2.5 HU compared to the supine position (Figure 2).

Each scanned region of the phantom trunk experienced a different pattern of CT number change as a function of the combination of vertical off-centring, localiser direction and phantom position (supine/prone). Unexpectedly, the ROI located at the heart (mid thorax) and near the trachea (upper thorax) did not display similar values of CT number change with vertical off-centring as with other ROIs. The maximum change recorded with ±100 mm off-centring for ROIs located on the heart and near the trachea was 13.9 and 4.3 HU, respectively, compared to 33.6 HU for other inferiorly located ROIs at the same scanned slice using 120 kVp. The CT number nearest the spine for all anatomical areas (upper thorax, mid thorax and abdomen) was shown to increase when the ROI was shifted vertically, away from the gantry iso-centre and decreased in proximity to the iso-centre. The phantom position was evidenced to have a significant difference on the CT numbers for the supine position (M = 10.75, SD = 14.82) compared to the prone position (M = −10.85, SD = 19.52); t(125) = 7.86, *p* < 0.001. Using 120 kVp and ±100 mm off-centring, the phantom positions caused the CT number to vary by 29.6 HU and 25.9 HU with the 0° localiser and 20.1 HU and 17.5 HU with the 180° localiser for both the upper and mid thorax regions, respectively.

The absolute differences in CT number between the farthest two off-centring points (100 mm above and below the gantry iso-centre) were larger for the inferior ROIs in the supine position, while the differences at the superior ROIs were higher in the prone position for both upper and mid thorax regions, as seen in Table 1. This behaviour was dissimilar for abdominal sections, in general, whereby the inferior ROIs demonstrated a more considerable CT number change compared to the superior ROIs, (Table 1).

**Figure 3:**
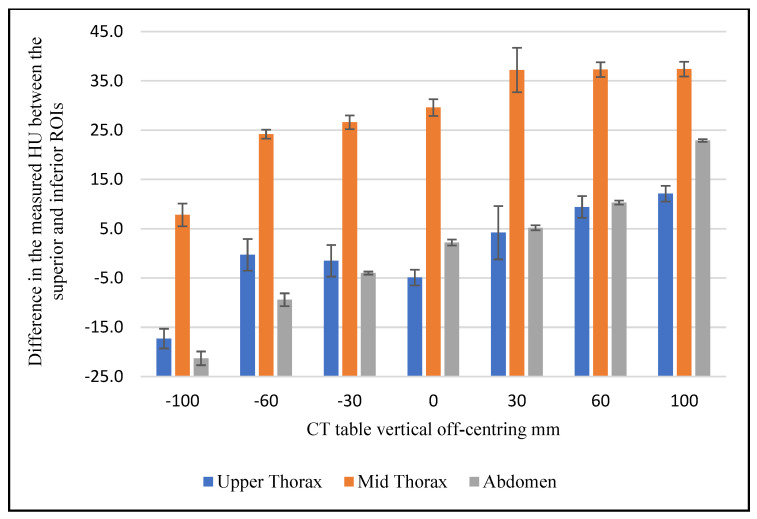
The difference in the mean CT number between the superior and inferior ROIs as a function of vertical off-centring for the upper thorax, mid thorax and abdominal phantom sections, at 120 kVp and 0° localiser.

**Figure 4:**
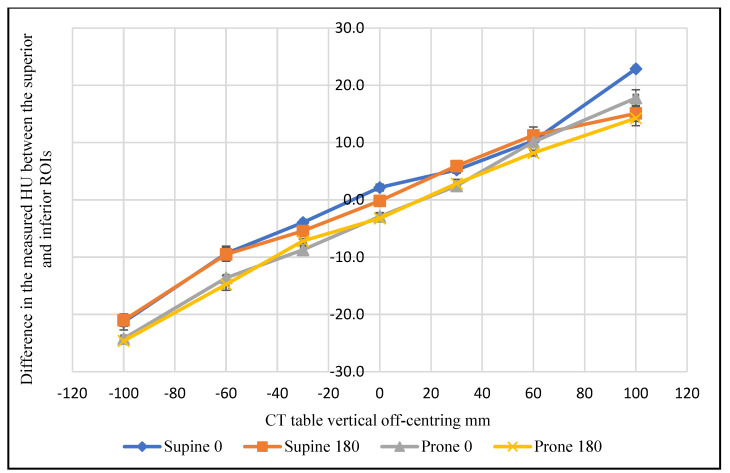
The difference in the mean CT number in the abdominal section between the superior and inferior ROIs as a function of vertical off-centring, phantom position, and localiser direction, at 120 kVp.

Figure 3 shows the difference between the mean of superior and inferior ROI measurements for the three scanned sections using 0° localiser and 120 kVp. This difference clearly varies across the scanned anatomical regions as the distance from the iso-centre increases. The CT number varied as a function of vertical off-centring by a maximum of 17.3 HU in the high thorax, 37.4 HU in the mid thorax, and 22.9 in the abdominal region. Figure 4 evidences that the mean difference in the CT number between the superior and inferior ROIs was influenced by the phantom position (supine and prone) for the abdominal section. The measured value for the same ROI varied by a maximum of 5.1 HU as a function of phantom position at 120 kVp.

**Figure 5:**
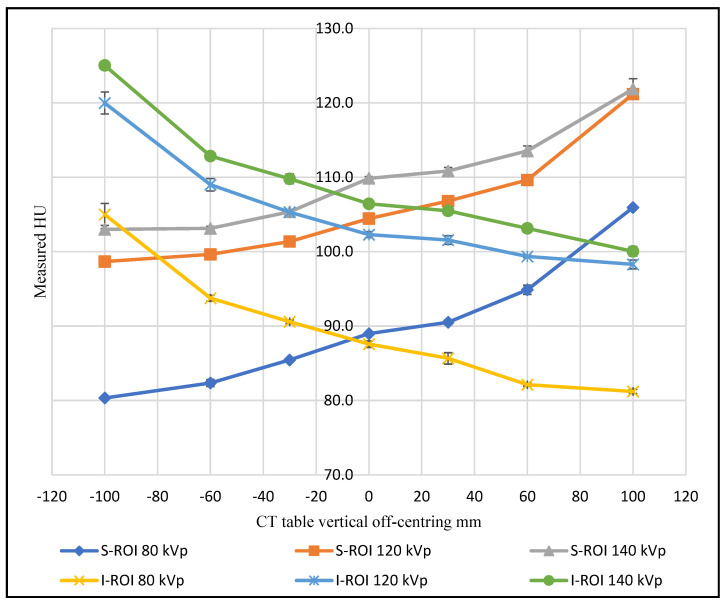
The CT number change of superior and inferior ROIs in the abdominal scans as a function of phantom vertical off-centring using different tube voltages.

Varying the tube voltage produced a difference in the measured CT number for the soft tissue elements of the phantom. Generally, as tube voltage increased, the CT number increased at the gantry iso-centre. The results also demonstrated that the tube voltage influenced the CT number change when the phantom was shifted away from the iso-centre, with different phantom positions and localiser directions (Figure 5). There was no clear trend in the data with values seen to be influenced by the scanned region (upper and mid thorax) and whether the ROI was located near soft tissue, bone or air.

**Figure 6:**
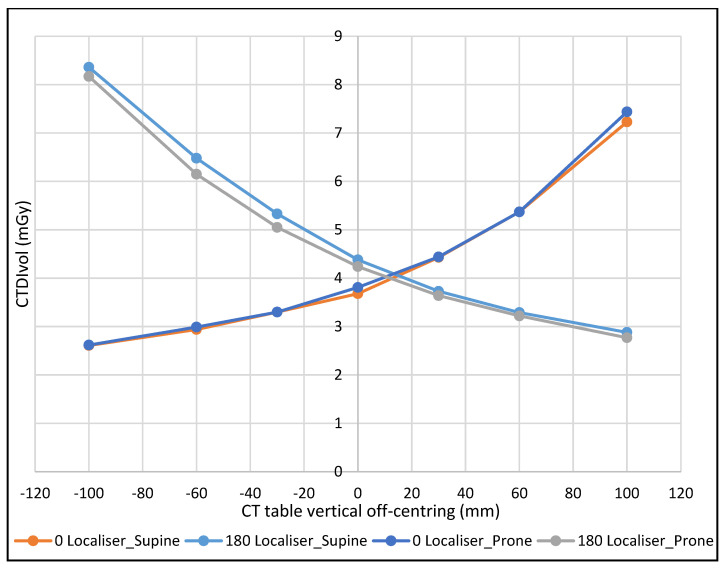
The effect of CT table off-centring on CTDI_vol_ with different phantom positions and localiser directions at 120 kVp.

As expected, the dose variation was lowest when the phantom was positioned at −100 mm from the gantry iso-centre with the 0° localiser, and the greatest with the 180° localiser at the same table height, with the converse true at +100 mm. The percentage dose variation, using 120 kVp, ranged from 69% to 195% with the 0° localiser and 65% to 193% with the 180° localiser when the phantom was in the prone position. Whereas with supine position, the percentage ranged from 71% to 196% with the 0° localiser, and 66% to 191% with the 180° localiser, see Figure 6.

## 4. Discussion

CT number variation as a function of vertical off-centring was demonstrated to be affected by the scanned area, ROI location, localiser direction, and phantom position (supine or prone). In the scanned abdominal region, the CT number decreased inside the ROI when it moved closer to the gantry iso-centre and increased when it moved away. These findings were consistent with the previous results of Hsieh, who found that the measured average CT number inside an ROI at the centre of a water phantom increased as it was vertically raised away from the gantry iso-centre to +120 mm [22], which can mostly be explained by the effect of the bowtie filter. Likewise, Szcykutowicz et al. reported in their experiment using an anthropomorphic phantom that CT numbers changed more than 20 HU for some ROIs when the phantom was shifted ±100 mm vertically from the iso-centre [13].

The localiser direction was also demonstrated to influence CT number change, with the closer the ROI to the localiser direction (X-ray tube), the larger the CT number measured, especially at the two farthest vertical off-centring levels (±100 mm). When the phantom was supine, the abdominal area showed a change in the CT number in the superior ROIs of 17 HU with the 0° localiser compared to the value at the iso-centre, while this difference was reduced to 8 HU with 180° localiser.

The measured CT numbers for the upper and mid thorax exhibited a different trend compared to the abdominal section, which was similarly reported by Szcykutowicz et al. However, the phantom position had more influence on the CT number at the upper and mid thorax regions with vertical off-centring compared to the abdomen. Beam-hardening artifacts and the reconstruction algorithm used are known to contribute strongly to these trends which are primarily influenced by the location of measured ROIs in relation to air-filled spaces and bone structures [13]. Moreover, the phantom position had a greater influence on the CT number change at the upper thorax section compared to the mid thorax. This can be explained by considering the presence of air-filled space (lungs and trachea) within the scanned thorax and their influence on the imaging algorithm [13]. The change in the measured CT number near the spine was larger when the ROI was shifted away from the gantry iso-centre toward the localiser direction. This is due to increased attenuation as this area received less radiation when it was projected on the thinnest part of the bowtie filter [13]. Current findings confirm that there is a linear relationship between the difference in the CT numbers recorded between the superior and inferior ROIs and the vertical off-centring in the abdominal section of the phantom (Figure 3).

It is generally accepted that attenuation co-efficient for objects/materials is energy-dependent. The measured and calculated attenuation co-efficient of an object will decrease with increasing kVp, as the average photon energy will also increase. Equation (1) is a means of making HU energy independent.

(1) $$\mathrm{HU}=\frac{\mu_{\mathrm{tissue}}-\mu_{\mathrm{water}}\;}{\mu_{\mathrm{water}}}\times 1000$$

However, HU dependence on tube voltage has been widely investigated, confirming that HU tissue values differ as a function of tube voltage with scanners from various manufacturers [12,23]. Generally, it can be concluded that the CT number for most of the materials with densities similar to or less than water (for example, breast and adipose tissue) will increase as the tube voltage increases, while the CT number will increase as tube voltage decreases for materials with densities greater than water, such as bone tissue. This concurs well with the current findings.

As a function of vertical off-centring, the CT number change was found to be affected by the tube voltage settings. There was no clear trend for the tube voltage influence across the scanned regions. However, the CT number change is seen to vary more at low tube voltage settings in some measurements (Figure 5). This can be explained by the fact that photoelectric absorption is more dominant at low tube voltages in contrast to Compton scattering, which predominates as the photon energy increases [24].

The current findings regarding CT number variability could confound the validity of quantitative CT for tissue lesion characterisation and subsequent clinical decision-making. Based on the results of this study, CT number uncertainty caused by inappropriate positioning may directly impact the assessment of adrenal and renal lesions, which are located posteriorly in the abdomen. Similarly, the demonstrated variation in the CT number between the superior and inferior ROIs through a relatively large abdominal organ, such as the liver, would have clinical significance. Importantly, patient off-centring has been demonstrated to influence changes in the CT number for the same tissue in the reconstructed images.

Incremental table off-centring was seen to cause variation of the scanner’s CTDIvol. The closer the proximity of the phantom to the X-ray source, the greater the magnification in the localiser radiograph and vice versa. This impacts the radiation output, which will vary depending on the apparent phantom size in the acquired localiser.

There is limited published work on the relationship between radiation dose and CT numbers [25]. The current research has demonstrated a strong, positive correlation between dose and CT numbers change, (r = 0.969, *p* < 0.001); 95% CI (0.93, 0.99) both demonstrating the same variation trend (Figure 2 and Figure 6).

### Limitations

A limitation of this study is that only one bowtie filter size (large), one average phantom size and one scanner model from one manufacturer was evaluated. Furthermore, the impact of vertical off-centring on image quality was not investigated.

## 5. Conclusions

Localiser direction, phantom position, the anatomy examined, and the location of the ROI has been demonstrated to affect CT number change as a function of vertical-off-centring. This highlights the importance of radiographer education in optimal patient centring for CT examinations and the need for radiologists to have an in-depth knowledge of CT number variation in clinical decision-making, especially when they are dependent on CT number accuracy for tissue lesion characterisation. Further research to examine the impact of phantom size on CT number variation as a function of vertical off-centring across multiple vendors is needed to validate this study.

## Figures and Tables

**Table 1:** The absolute computed tomography (CT) number difference of the ROI between the 100 mm off-centring points above and below the gantry iso-centre for 0° and 180° localisers and supine and prone phantom positions.

		80 kVp		120 kVp		140 kVp	
		S.ROI ^a^	I.ROI ^b^	S.ROI	I.ROI	S.ROI	I.ROI
0° localiser/Supine position	Upper Thorax	29	10.8	4.3	33.6	0.5	27.9
	Mid Thorax	6.1	28.3	3.5	26.0	4.2	29.1
	Abdomen	25.6	23.8	22.5	21.7	18.9	25.0
180° localiser/Supine position	Upper Thorax	8.7	27.6	1.4	23.0	1.5	21.0
	Mid Thorax	2.8	43.3	2.5	31.4	1.6	29.3
	Abdomen	14.3	23.5	12.0	24.8	12.4	23.0
0° localiser/Prone position	Upper Thorax	17.7	0.9	6	4.0	19.5	7.4
	Mid Thorax	33.1	0.1	23.4	0.1	21.0	10.4
	Abdomen	22.5	30.8	18.7	23.3	17.4	28.5
180° localiser/Prone position	Upper Thorax	10.9	7.0	17.2	2.9	12.2	4.0
	Mid Thorax	15.9	15.4	14.8	13.9	13.5	9.9
	Abdomen	14.6	29.0	11.7	27.2	12.4	32.1

^a^ Superior ROI, ^b^ Inferior ROI.

## Data Availability

Available from the corresponding author.
